# Heterozygous Deficiency of Endoglin Decreases Insulin and Hepatic Triglyceride Levels during High Fat Diet

**DOI:** 10.1371/journal.pone.0054591

**Published:** 2013-01-15

**Authors:** Daniel Beiroa, Amparo Romero-Picó, Carmen Langa, Carmelo Bernabeu, Miguel López, José M. López-Novoa, Ruben Nogueiras, Carlos Diéguez

**Affiliations:** 1 Department of Physiology, School of Medicine-CIMUS – Instituto de Investigaciones Sanitarias (IDIS), CIBER Fisiopatologia de la Obesidad y Nutricion (CIBERobn), University of Santiago de Compostela, Santiago de Compostela, A Coruña, Spain; 2 Centro de Investigaciones Biológicas, Consejo Superior de Investigaciones Científicas (CSIC), and Centro de Investigación Biomédica en Red de Enfermedades Raras (CIBERER), Madrid, Spain; 3 Renal and Cardiovascular Physiopathology Unit, Department of Physiology and Pharmacology, University of Salamanca and Instituto de Investigaciones Biomédicas de Salamanca (IBSAL), Campus Miguel de Unamuno, Salamanca, Spain; University of Cordoba, Spain

## Abstract

Endoglin is a transmembrane auxiliary receptor for transforming growth factor-beta (TGF-beta) that is predominantly expressed on proliferating endothelial cells. It plays a wide range of physiological roles but its importance on energy balance or insulin sensitivity has been unexplored. Endoglin deficient mice die during midgestation due to cardiovascular defects. Here we report for first time that heterozygous endoglin deficiency in mice decreases high fat diet-induced hepatic triglyceride content and insulin levels. Importantly, these effects are independent of changes in body weight or adiposity. At molecular level, we failed to detect relevant changes in the insulin signalling pathway at basal levels in liver, muscle or adipose tissues that could explain the insulin-dependent effect. However, we found decreased triglyceride content in the liver of endoglin heterozygous mice fed a high fat diet in comparison to their wild type littermates. Overall, our findings indicate that endoglin is a potentially important physiological mediator of insulin levels and hepatic lipid metabolism.

## Introduction

Endoglin (Eng) is a transmembrane homodimeric glycoprotein (180 kDa) identified in human vascular endothelial cells where it is highly expressed [1]. Eng is also expressed in many other cells types including smooth muscle cells, mesangial cells, fibroblasts, hepatocytes, and keratinocytes [2]. Eng functions as a non-signaling coreceptor of the transforming growth factor beta (TGF-β) modulating its responses [2], [3].

Eng modulates processes mainly related to vascular physiology and pathophysiology [2]. Eng plays a key role in endothelium-mediated vascular reactivity as it regulates the expression of endothelial nitric oxide synthase (eNOS), and consequently the synthesis of nitric oxide (NO) [4]–[6] and the expression of cyclooxygenase 2 (COX-2) [7]. Eng expression increases during alterations in vascular structure and function as during embryogenesis, inflammation and wound healing [8] and it is necessary for endothelial cell survival during hypoxia [9]. Eng is required for normal angiogenesis during fetal development as Eng null embryos die at 10–11.5 days due to vascular and cardiac abnormalities [9]–[11]. Eng also modulates various processes involved in the regulation of angiogenesis in the adult including tumor growth [12]–[16]. Furthermore, Eng appears involved in the vascular repair carried out by blood mononuclear cells [17] and is associated to hypertension during pregnancy [18], [19].

Mutations in the endoglin gene leading to endoglin haploinsufficiency are the cause of the Hereditary Hemorrhagic Telangiectasia (HHT) type 1 [20], [21]. Interestingly, gene expression fingerprinting of blood outgrowth endothelial cells demonstrated that compared to healthy subjects, HHT1 patients show 20% of deregulated genes (upregulated or down regulated) that are involved in metabolic homeostasis [22]. Supporting the link between Eng and metabolism, a relationship between plasma levels of Eng and glycemia was recently found in diabetic patients [23]. In addition, endoglin deficiency is related to endothelial dysfunction [2] and there is a clear association between endothelial dysfunction and alterations in glucose metabolism or metabolic syndrome [24], [25]. In spite of these evidences, the endogenous role of Eng on energy balance or glucose metabolism is largely unknown. The present study is the first one aimed to investigate the metabolic phenotype of mice haploinsufficient for Eng (*Eng^+/−^*) in normal conditions or when challenged with high fat diet.

## Materials and Methods

### Animals

Generation and genotyping of *Eng^+/−^* mice on a C57Bl/6 background was previously described [11], [26]. Mice were kept in ventilated rooms, in a pathogen-free facility under conditions of controlled temperature (23°C), humidity (50%) and illumination (12-hour light/12-hour dark cycle). All studies were performed in parallel in *Eng^+/−^* and *Eng^+/+^* littermate male mice of 4–6 months of age (20–25 g). After weaning, mice were fed a standard chow diet, and after 8 weeks, the diet was changed to high fat diet (HFD, Research Diets 12451; 45% fat, 4.73 kcal/g, Research Diets, New Brunswick, NJ) during 16 weeks. All animal procedures performed were approved by the University of Salamanca Animal Care and Use Committee and by the Animal Committee at the University of Santiago de Compostela. All the experiments were performed in agreement with the Rules of Laboratory Animal Care and International Law on Animal Experimentation.

### Determination of body composition and energy balance

Whole body composition was measured using NMR imaging (Whole Body Composition Analyzer; EchoMRI, Houston, TX). Animals were monitored in a custom 12-cage indirect calorimetry, food intake and locomotor activity monitoring system (TSE LabMaster, TSE Systems, Germany) as previously described [27], [28]. Mice were acclimated for 48 hr to the test chambers and then were monitored for an additional 48 hr. Data collected from the last 48 hr was used to calculate all parameters for which results are reported.

### Quantitative reverse transcriptase PCR (qRT-PCR) analysis

RNA was extracted using Trizol® reagent (Invitrogen) according to the manufacturer's instructions and two micrograms of total RNA were used for each RT reaction and cDNA synthesis was performed using SuperScript™ First-Strand Synthesis System (Invitrogen) and random primers as previously described [29]. Negative control reactions, containing all reagents except the sample were used to ensure specificity of the PCR amplification. For the analysis of gene expression we used real-time reverse-transcription polymerase chain reaction (RT-PCR) analyses performed in a fluorescent temperature cycler (TaqMan®; Applied Biosystems; Foster City, CA, USA) following the manufacturer's instructions [29], [30]. Five hundred ng of total RNA were used for each RT reaction. The PCR cycling conditions included an initial denaturation at 50°C for 10 min followed by 40 cycles at 95°C for 15 sec; 60°C for 1 min. The oligonucleotide specific primers and probes were: G6Pase Fw 5′-CCA GGT CGT GGC TGG AGT CT-3′, Rv 5′-TGT AGA TGC CCC GGA TGT G-3′, 5′-FAM-CAG GCA TTG CTG TGG CTG AAA CTT TCA G-TAM-3′; and PEPCK1 Fw 5′-CCA CAG CTG CTG CAG AAC AC-3′, Rv 5′-GAA GGG TCG CAT GGC AAA-3′, 5′-FAM-AGG GCA AGA TCA TCA TGC ACG ACC C-TAM-3′. For the analysis of the data, the input value of the gene expression was standardized to the 18S value for each sample of each group and was expressed compared with the average value for the control group.

### Western blot analysis

Western blots were performed as previously described [28], [31]. Briefly, total protein lysates from liver (20 µg), muscle (20 µg), and WAT (15 µg) were subjected to SDS-PAGE, electrotransferred onto a polyvinylidene difluoride membrane and probed with antibodies against NFkB, PTEN, AKT, pAKT (Ser473), (Cell Signaling, Danvers, MA), and Glut4 (Santa Cruz Biotechnology, Santa Cruz, CA). Recombinant human endoglin tagged with the hemagglutinin (HA) epitope was detected with 12CA5 monoclonal antibody (Roche Diagnostics, Mannheim, Germany). As a loading control, monoclonal antibodies to β-actin (clone AC-15, Sigma) were used. For primary antibody detection we used horseradish peroxidase-conjugated secondary antibodies and chemiluminescence (Thermo Scientific). We used eight mice per group and the protein levels were normalized to β-actin for each sample.

### Glucose and insulin tolerance tests

Blood glucose levels were measured with an Accucheck glucometer (Roche) after an intraperitoneal injection of either 2 mg/g D-glucose (Sigma) or 0.75 U/kg insulin (Sigma-Aldrich) [32]. Area under the curve (AUC) values were determined and data were analyzed with one-way ANOVA and post-hoc analysis as previously described [27]. GTT and ITT AUC curves were also analyzed with two-way ANOVA using as factors genotype and diet.

### TG content in liver

The extraction procedure for tissue TG was adapted from methods described previously [28]. Livers (aprox 200 mg) were homogenized for 2 min in ice-cold chloroform-methanol (2∶1, vol/vol). TG were extracted during 5-h shaking at room temperature. For phase separation, H_2_SO_4_ was added, samples were centrifuged, and the organic bottom layer was collected. The organic solvent was dried using a Speed Vac and redissolved in chloroform. TG (Randox Laboratories LTD, UK) content of each sample was measured in duplicate after evaporation of the organic solvent using an enzymatic method.

### Levels of plasma metabolites and hormones

Plasma glucose was measured by the glucose oxidase method (Glucose and Triglyceride Spinreact, Spain). Plasma nonesterified fatty acids (NEFA) concentrations were determined using a kit from Wako (US); triacylglycerol (TG) and cholesterol were determined using a kit from Randox Laboratories (LTD, UK). Plasma insulin levels were measured by a previously described RIA [27].

### Data Analysis and Statistics

Values are plotted as the mean ± SEM for each genotype. Statistical significance was determined by Student's t -test. A P value less than 0.05 was considered statistically significant.

## Results

### *Eng^+/−^* mice fed a standard diet do not show metabolic alterations

**Figure 1:**
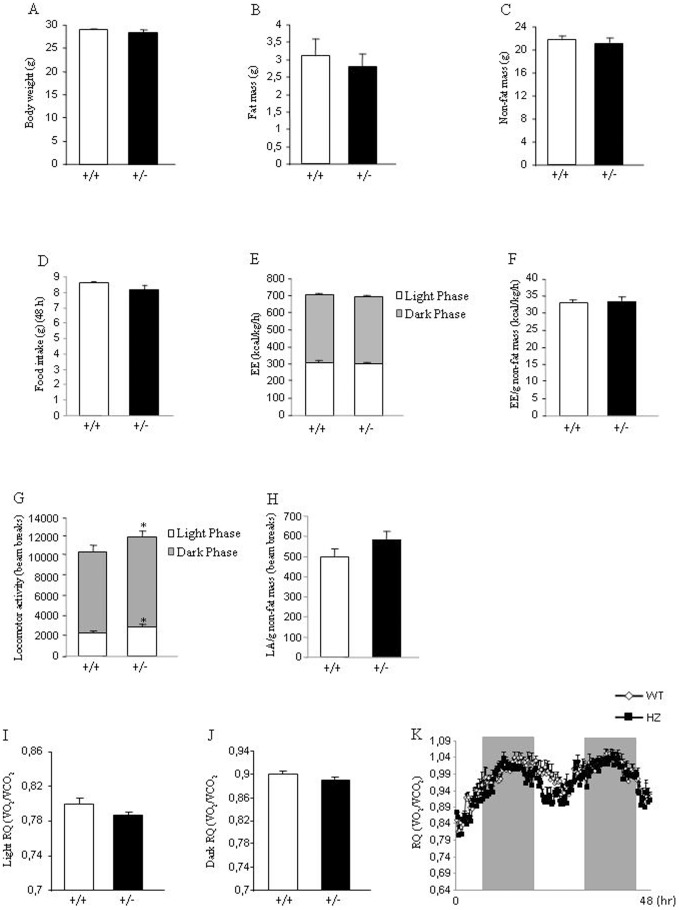
Body weight, body composition, food intake, and metabolic parameters in mice fed a standard diet. Body weight (A), fat mass (B), non-fat mass (C), food intake (D), total energy expenditure (E), energy expenditure corrected by non-fat mass (F), total locomotor activity (G), locomotor activity corrected by non-fat mass (H), respiratory quotient during light phase (I), respiratory quotient during dark phase (J), and 48 h profile of RQ (K) in 8-week male wild type and endoglin heterozygous mice fed a standard diet. Measurements were done during 48 h. n = 6–8. *p<0.05.

Age-matched male WT and *Eng^+/−^* mice of 4 weeks of age were maintained on standard diet for 8 weeks to assess their metabolic phenotypes. No body weight differences were found between both genotypes (Figure 1A). Consistently, body composition (fat mass and non fat mass) (Figure 1B and 1C) and food intake (Figure 1D) were not altered. Indirect calorimetry was used to determine locomotor activity, energy expenditure and respiratory quotient (RQ). Energy expenditure remained unchanged when WT and *Eng^+/−^* mice were fed a standard diet (Figure 1E–1F). Although a slight but significant increase in the locomotor activity of *Eng^+/−^*, as compared to WT mice, was observed (Figure 1G), this increase was not found when locomotor activity was corrected by grams of non-fat mass (Figure 1H). In addition, the RQ did not show any statistical difference during the light (Figure 1I and 1K) or dark phase (Figure 1J and 1K).

### Glucose homeostasis in *Eng^+/−^* mice fed a standard diet

**Figure 2:**
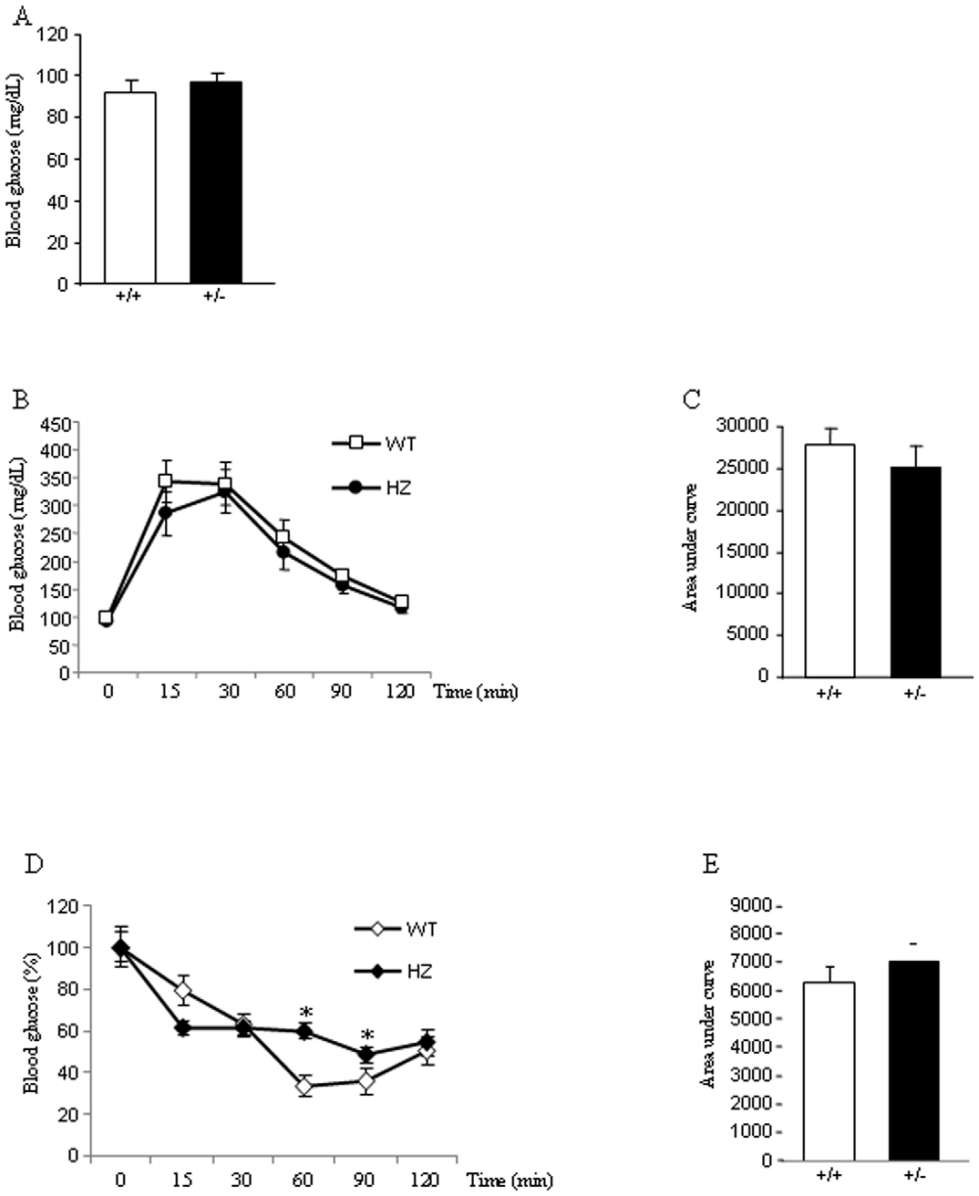
Glucose homeostasis and insulin sensitivity in mice fed a standard diet. Basal glucose levels (A), glucose tolerance test (B), respective area under the curve (C), insulin tolerance test (% of glucose levels represented against t0) (D), and respective area under the curve (E) in 8-week male wild type (WT) and endoglin heterozygous (HZ) mice fed a standard diet. n = 6–8. *p<0.05.

Next, we assessed key parameters of glucose homeostasis in 8 weeks old WT and *Eng^+/−^* mice fed a standard diet. This analysis revealed unaltered fasting blood glucose concentrations in *Eng^+/−^* mice compared to controls fed a chow diet (Figure 2A). When *Eng^+/−^* mice, fed a chow diet, were subjected to intraperitoneal glucose tolerance tests (ipGTT) after an overnight fasting, we did not find any alteration in the tolerance to glucose as compared to WT littermates (Figure 2B–2C). Furthermore, insulin tolerance tests (ITT) after an overnight fasting showed that *Eng^+/−^* mice had increased glucose levels after 60 and 90 min of insulin injection (Figure 2D). When integrated (area under the curve) glucose levels in *Eng^+/−^* mice fed a chow diet failed to show significant differences compared with WT control mice (Figure 2E).

### *Eng^+/−^* mice fed a HFD show normal body weight and metabolic phenotype

**Figure 3:**
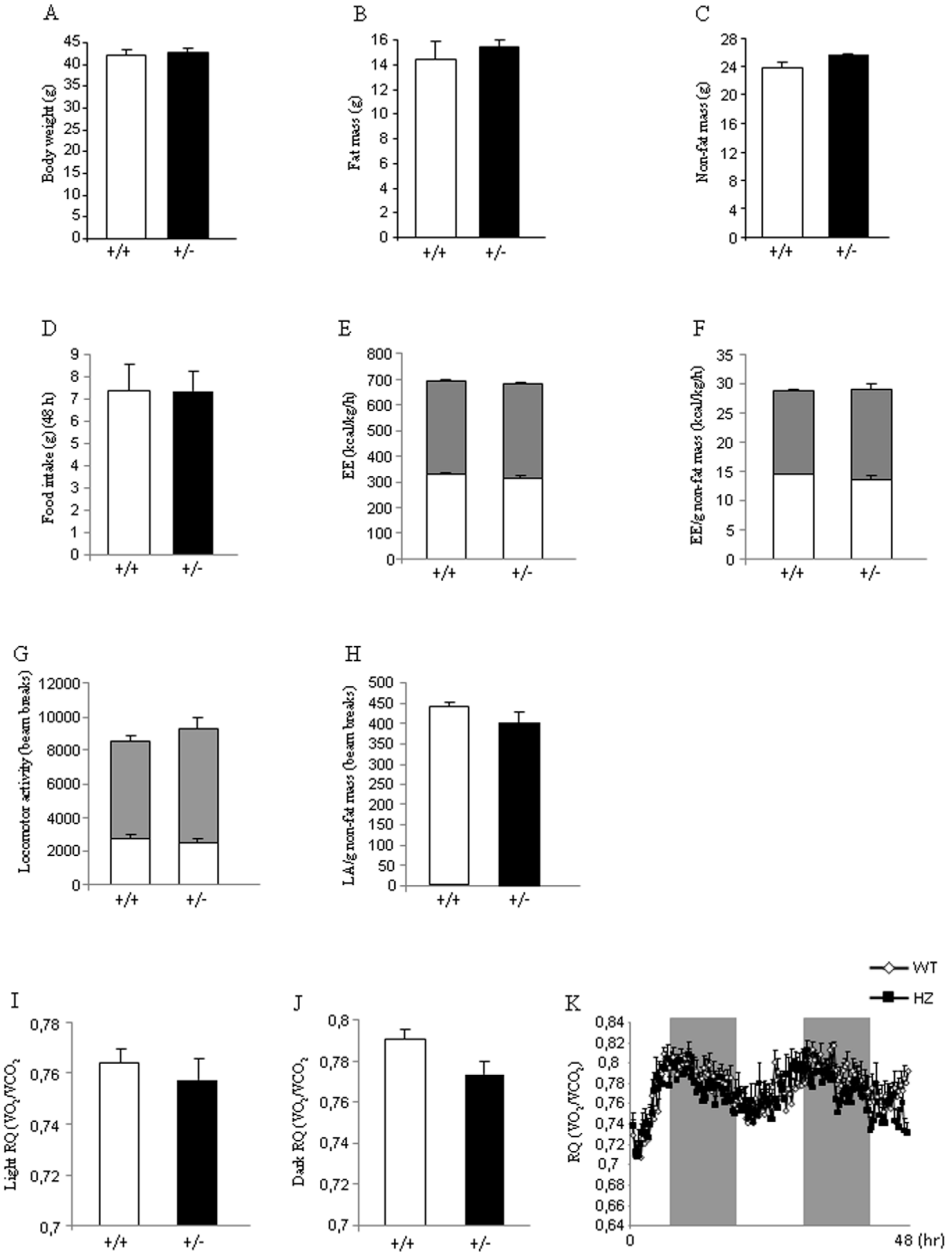
Body weight, body composition, food intake, and metabolic parameters in mice fed a high fat diet. Body weight (A), fat mass (B), non-fat mass (C), food intake (D), total energy expenditure (E), energy expenditure corrected by non-fat mass (F), total locomotor activity (G), locomotor activity corrected by non-fat mass (H), respiratory quotient during light phase (I), respiratory quotient during dark phase (J), and 48 h profile of RQ (K) in male wild type and endoglin heterozygous mice fed a high fat diet for 16 weeks. Measurements were done during 48 h. n = 6–8.

Age-matched male WT and *Eng^+/−^* mice of 8 weeks of age were maintained on HFD (45% kcal fat, 4.73 kcal/g) for 16 wk to assess their metabolic phenotypes. No differences in body weight were found between *Eng^+/−^* and WT mice when they were fed a HFD (Figure 3A). Consistently, body composition analysis with quantitative NMR revealed that *Eng^+/−^* mice fed a HFD gained a similar amount of fat (Figure 3B) and non-fat mass (Figure 3C) compared to WT mice after 16 weeks with HFD. Food intake was also very similar between both genotypes (Figure 3D). Energy expenditure remained unchanged when WT and *Eng^+/−^* mice were fed a HFD (Figure 3E–3F). No changes were observed in the total locomotor activity between WT and *Eng^+/−^* mice (Figure 3G). A similar result was observed when locomotor activity was corrected by grams of non-fat mass (Figure 3H). The RQ did not show any statistical difference during the light (Figure 3I and 3K) or dark phase (Figure 3J and 3K).

### Glucose homeostasis in *Eng^+/−^* mice fed a HFD

**Figure 4:**
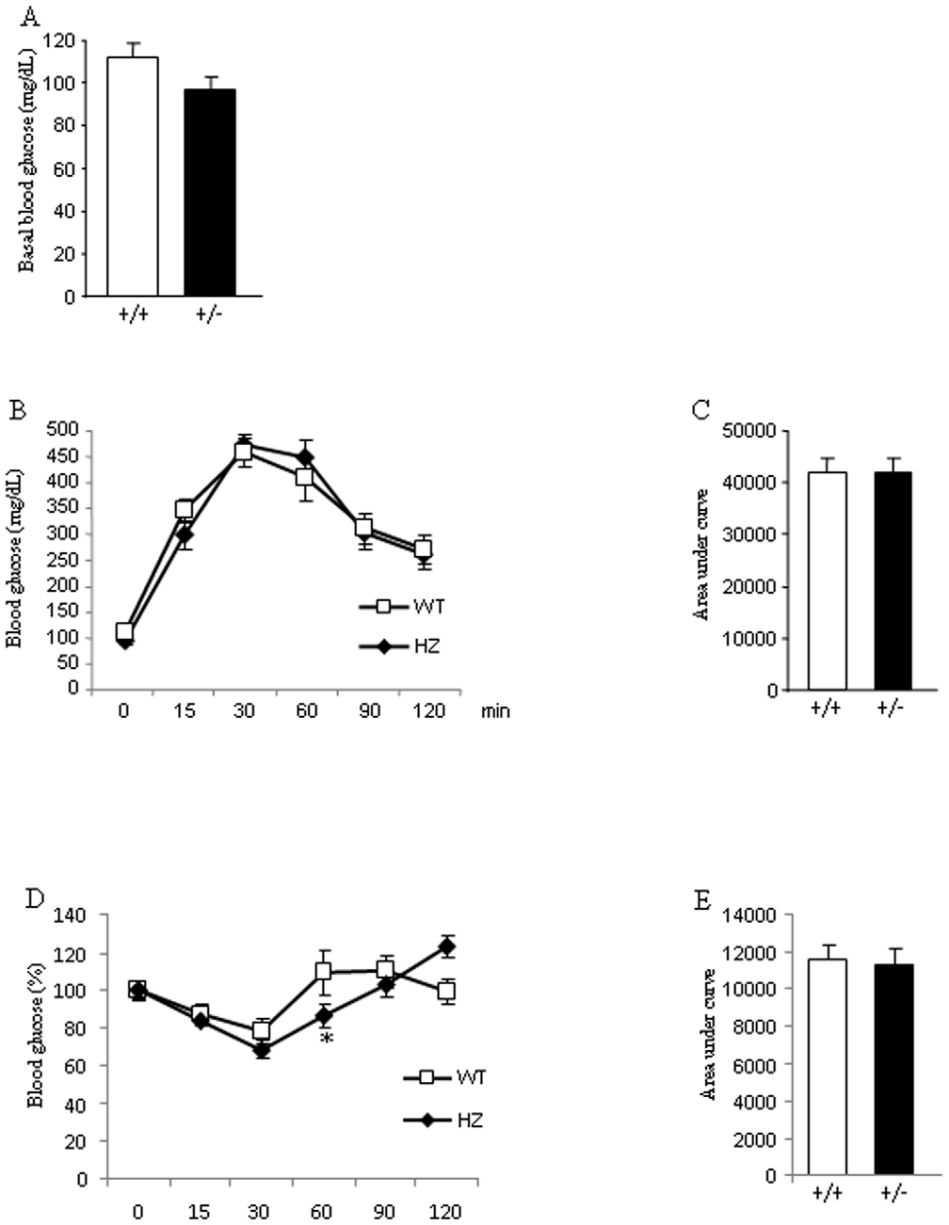
Glucose homeostasis and insulin sensitivity in mice fed a high fat diet. Basal glucose levels (A), glucose tolerance test (B), respective area under the curve (C), insulin tolerance test (% of glucose levels represented against t0) (D), and respective area under the curve (E) in male wild type (WT) and endoglin heterozygous (HZ) mice fed a high fat diet for 16 weeks. n = 6–8. *p<0.05.

After an overnight fast, plasma glucose levels did not differ between WT and *Eng^+/−^* mice fed a HFD (Figure 4A). After an overnight fast, a GTT failed to reveal significant differences in glucose tolerance between both genotypes (Figure 4B–4C). To further investigate the endogenous role of Eng in the control of glucose metabolism, mice were subjected to an ITT after an overnight fast. After 60 min of insulin injection, glucose values in *Eng^+/−^* mice were lower than in WT mice, but no differences were found at the other time points (Figure 4D). When integrated (area under the curve) glucose levels in *Eng^+/−^* mice fed a HFD failed to show any significant differences when compared with WT control mice (Figure 4E).

**Figure 5:**
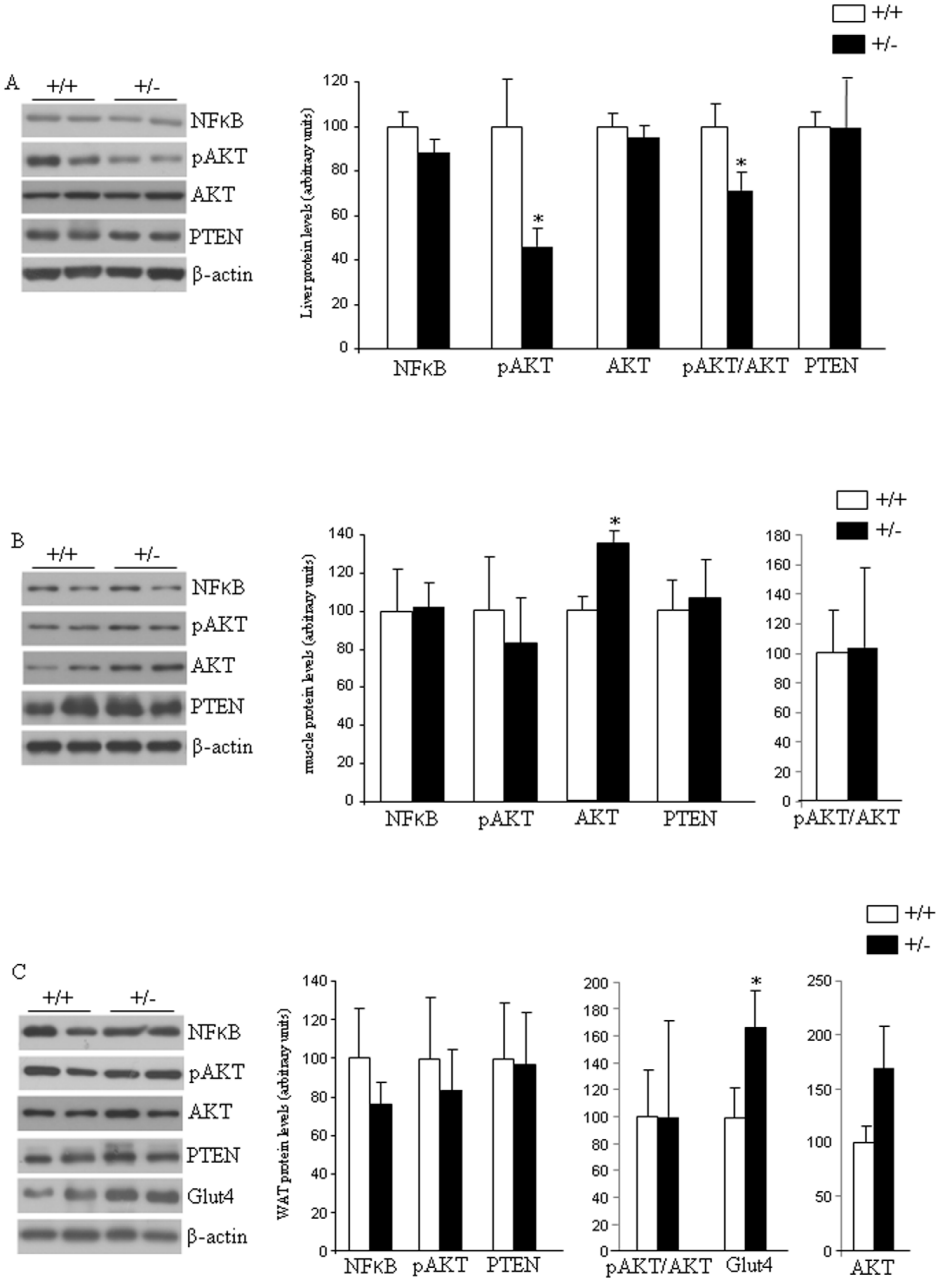
Insulin signaling and glucose uptake in mice fed a high fat diet. Protein levels of NFkB, pAKT, AKT, and PTEN in the liver (A), and muscle (B) of male wild type and endoglin heterozygous mice fed a high fat diet for 16 weeks. Protein levels of NFkB, pAKT (Ser473), AKT, PTEN, and Glut4 in the white adipose tissue (C) of male wild type (+/+) and endoglin heterozygous (+/−) mice fed a high fat diet for 16 weeks. All the samples (+/+ and +/−) for each protein were analyzed within the same gel, and the lines represent splicings of the gels. n = 6–8. *p<0.05.

Next, we determined whether *Eng* interferes with the expression of insulin-signaling components by measuring transcription factor nuclear factor-kappaB (NFkB), pAKT, AKT, and phosphatase and tensin homolog (PTEN) protein levels in liver, skeletal muscle and adipose tissue samples. In the liver we found decreased levels of AKT phosphorylation (Ser473) and the ratio pAKT/AKT, whereas no changes were detected in NFkB, AKT or PTEN (Figure 5A). In muscle, protein levels of these four factors were unaffected by the partial lack of *Eng* (Figure 5B). In the white adipose tissue (WAT), we also failed to detect changes in protein levels of NFkB, pAKT, AKT, ratio pAKT/AKT, and PTEN (Figure 5C). However, we found increased levels of glucose transporter 4 (Glut4) in the WAT of *Eng^+/−^* mice fed a HFD in comparison to WT mice (Figure 5C).

**Figure 6:**
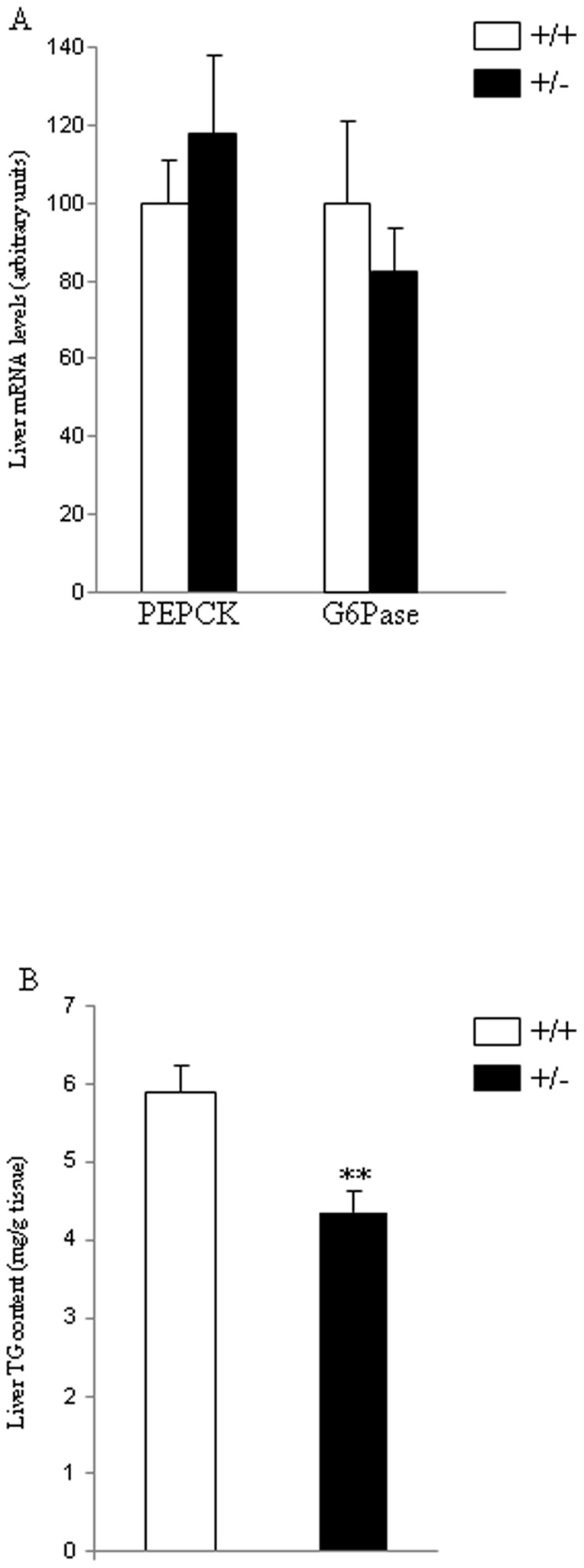
Hepatic glucose production in mice fed a high fat diet. mRNA levels of PEPCK and G6Pase (A) and triglyceride (TG) content (B) in the liver of male wild type and endoglin heterozygous mice fed a high fat diet for 16 weeks. 18S was used as an internal control. n = 6–8. **p<0.01.

Next, in order to test if the partial lack of *Eng* was affecting to hepatic glucose production, we measured the mRNA levels of hepatic glucose 6 phosphatase (G6Pase) and phosphoenolpyruvate carboxykinase (PEPCK) (Figure 6A), but failed to detect any change between *Eng^+/−^* mice fed a HFD and their littermates. However, triglyceride (TG) content in the liver of *Eng^+/−^* mice fed a HFD was significantly lower than in WT mice (Figure 6B).

**Figure 7:**
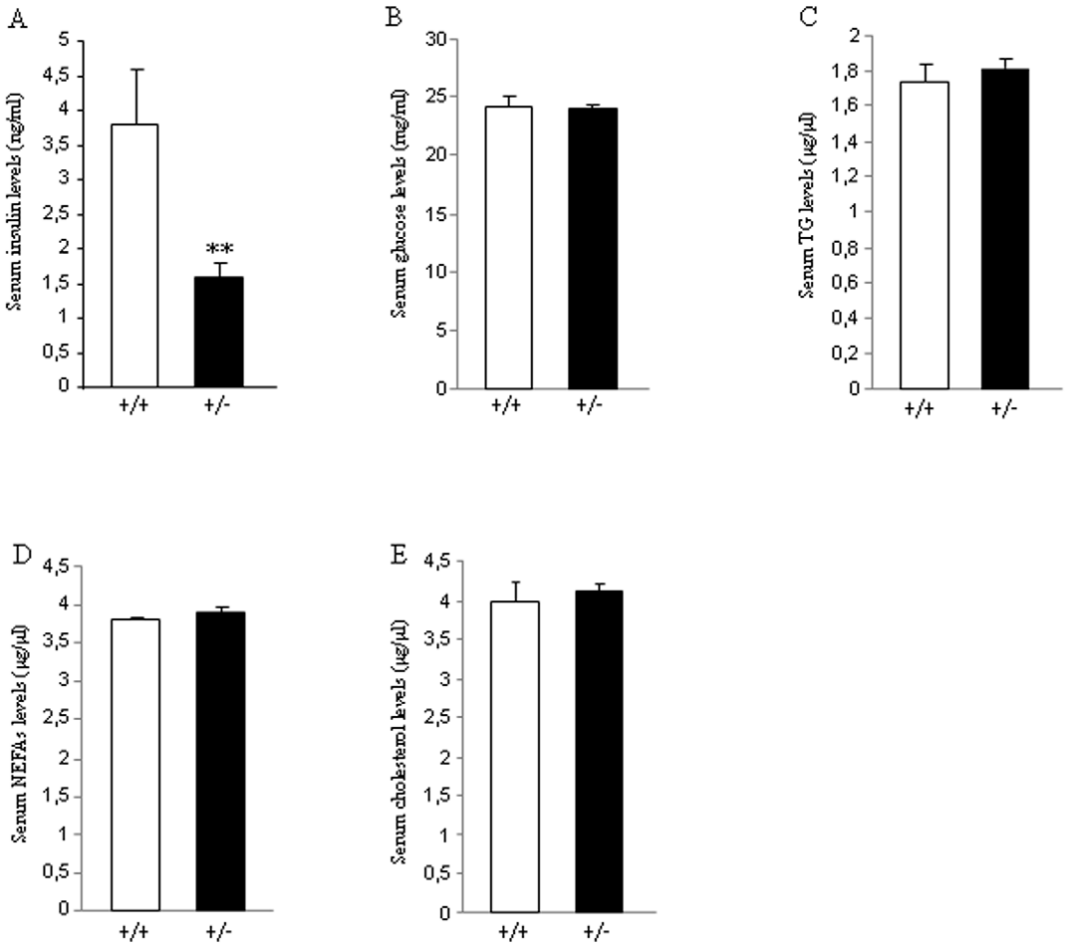
Levels of plasma metabolites and hormones in mice fed a high fat diet. Serum levels of insulin (A), glucose (B), TG (C), NEFAS (D), and cholesterol (E) in male wild type and endoglin heterozygous mice fed a high fat diet for 16 weeks. n = 6–8. **p<0.01.

Then, we measured serum levels of different metabolites and hormones when mice were sacrificed after 16 weeks on HFD. We found that the partial lack of *Eng* caused a significant decrease in insulin (Figure 7A), but no changes were detected in glucose (Figure 7B), TG (Figure 7C), NEFAs (Figure 7D) or cholesterol (Figure 7E) in WT or *Eng^+/−^* mice fed ad libitum.

## Discussion

Mice lacking Eng (*Eng^−/−^)* die during the embryonary phase due to defective angiogenesis [11], [33], but *Eng^+/−^* mice are viable, breed normally and represent a useful tool to assess the physiological role of endoglin [34]. Here we demonstrate that the partial lack of endoglin in *Eng^+/^* mice does not lead to significant changes in body weight, adiposity or food intake. Since HFD induces non-alcoholic fatty liver and causes hyperinsulinemia in mice, a remarkable observation is that after 16 weeks on HFD, *Eng^+/−^* mice showed decreased hepatic triglyceride content and lower insulin levels compared with WT mice.

Eng has emerged as a glycoprotein related with multiple biological actions including angiogenesis, vascular physiology, preeclampsia or cancer. Endoglin deficiency causes a decrease in NO synthesis and the subsequent endothelial dysfunction [2], a process usually associated with altered glucose metabolism and metabolic syndrome [24], [25]. However, to our knowledge, the potential role of Eng mediating energy homeostasis has been unexplored. Our current findings demonstrate that the heterozygous deficiency of Eng does not cause any important alteration in metabolic parameters affecting body weight regulation. Energy expenditure, locomotor activity, respiratory quotient or feeding behaviour were similar in WT and *Eng^+/−^* mice. Importantly, the lack of metabolic phenotype was observed when mice were fed a standard diet or HFD, indicating that endogenous Eng does not seem to be an important player in these metabolic alterations even though mice were challenged to undergo a pathophysiological condition.

Plasma soluble Eng levels have been reported to be positively correlated with basal glycemia in patients with diabetes and hypertension, and with glycated haemoglobin in all patients with diabetes [23]. Therefore, we also investigated the potential relevance of endogenous Eng in glucose metabolism. Our findings indicate that *Eng^+/−^* mice fed a standard diet did not show relevant changes in insulin sensitivity in comparison to their control littermates. When mice were challenged with HFD, which increases the risk of obesity and insulin resistance, we observed that insulin levels were significantly decreased when compared to WT mice fed under the same diet. However, insulin sensitivity was similar between *Eng^+/−^* and WT mice (Figure 4). Overall, previous data obtained in humans [23] and our current findings in rodents indicate that Eng seem to play an important role in the control of insulin levels, and heterozygous deficiency of Eng decreases HFD-induced hyperinsulinemia. It is important to highlight that this decrease in insulin levels is independent of changes in body weight or adiposity, suggesting that Eng might play a direct role on insulin synthesis and/or secretion. The PI3K/Akt pathway is one of the major downstream targets of the insulin pathway and is negatively regulated by phosphatase and tensin homologue deleted on chromosome 10 (PTEN) [34]. The activation of the PI3K/Akt pathway inhibits the release of soluble Eng from endothelial cells [35]. Conversely, the inhibition of the PI3K/Akt pathway, by overexpression of PTEN stimulates soluble Eng release from endothelial cells [35]. Given the link between Eng and these signaling proteins, we investigated the possibility that PTEN or Akt levels were altered in *Eng^+/−^* mice. We detected a significant decrease of pAkt levels in the liver of *Eng^+/−^* mice versus WT mice fed a HFD, but not in muscle or WAT. The inverse correlation between endoglin expression and activation of the survival route of Akt in the liver fits well with the anti-apoptotic effect and the active role in endothelial cell proliferation of endoglin [2], [9]. Furthermore, PTEN protein levels also remained unmodified between both genotypes. Taken together, these findings suggest that PTEN and Akt are differentially modulated by the partial lack of Eng, and that Akt is regulated in a tissue-specific manner. Cell culture and animal studies have also related NF-κB activity in the pathogenesis of insulin signalling [36]. We failed to detect any significant change in NF-κB protein levels in the liver, muscle or WAT between WT and *Eng^+/−^* mice, indicating that this transcription factor was not regulated by *Eng*.

At the cellular level, insulin stimulates glucose uptake by inducing the translocation of the glucose transporter 4 (GLUT4) from intracellular storage sites to the plasma membrane, where the transporter facilitates the diffusion of glucose into muscle and adipocytes [37]. Therefore, we assessed the protein levels of the glucose transporters in WAT, and found that Glut4 protein levels were significantly higher in *Eng^+/−^* mice fed a HFD when compared to their control littermates. The importance of GLUT4 expression for maintaining glucose homeostasis and insulin sensitivity has been extensively addressed in different animal models [38], and its essential role is reflected by the phenotype caused by the deficiency or over-expression of GLUT4 in mice [39]. Since *Eng^+/−^* mice present reduced insulin levels under HFD, the higher levels of Glut4 detected in the WAT of these mice could be a compensatory mechanism to the lower insulin levels. Another key aspect on diet-induced obesity is the increased amount of fatty acids in the liver [40]. Total hepatic TG content was lower in *Eng^+/−^* mice than in control mice fed a HFD. Overall, our data might suggest a defect in insulin production in beta cells from *Eng^+/−^* mice fed a HFD. Further studies using isolated islets will be necessary to clarify this aspect. Indeed, we cannot rule out the possibility that other factors important for insulin might be also affected in *Eng^+/−^* mice. In this regard, a comparative gene expression analysis revealed that in endothelial cells from HHT1 patients, 20% of the deregulated genes (down or upregulated) respect to cells from healthy subjects were involved in general metabolism [22]. Among these genes it is worth mentioning the presence of several members of the solute carrier (SLC) protein family. This family contains over 300 membrane transport proteins, including the glucose transporters Glut-1 (*SLC2A1*) and Glut-4 (*SLC2A4*). Thus, in HHT1 cells, electroneutral cation-Cl cotransporter SLC12A2 (Na-K-Cl cotransporter), mitochondrial carrier SLC25A29 (mitochondrial carnitine/acylcarnitine carrier protein CACL), fatty acid transport protein SLC27A3 (fatty acid transport protein 3), nucleoside-sugar transporter SLC35A5 (UDP-sugar transporter protein) and basolateral iron transporter SCL40A1 (ferroportin 1) were downregulated, whereas nucleoside-sugar transporters SLC35B2 (3′-phosphoadenosine 5′-phosphosulfate transporter) and SLC35D3 (fringe connection-like protein 1) were upregulated respect to controls. In addition to the evident involvement of these carrier proteins in the general metabolism, some of them have been reported to be involved in insulin-dependent metabolic pathways [41]–[43], thus supporting the link between Eng and insulin. Further studies will be necessary to address this issue.

In summary, we conclude that Eng has a physiological role in the regulation of insulin levels and hepatic lipid content, particularly under challenged environmental conditions. The decreased insulin levels and lower hepatic lipid content seem to be independent of changes in body weight or adiposity. These findings expand our knowledge on the physiological effects controlled by Eng, and identify Eng as a potentially important physiological mediator of metabolism.
